# Percutaneous Nephrolithotomy: Challenges for a Novice Urologist

**DOI:** 10.1155/2020/5053714

**Published:** 2020-05-08

**Authors:** Ashish Chaurasia

**Affiliations:** Asian Institute of Medical Sciences, MIDC, Milap Nagar, Dombivli (East), Maharashtra 421203, India

## Abstract

PCNL is the treatment of choice for large renal stones. It is a procedure of expertise. It may look simpler when done by an experienced urologist; however, for a beginner, learning each and every step is very important. He should be well-versed about the difficulties faced at every step and know how to tackle them. This article focuses mainly on the intricacies faced by a trainee during the procedure and how to troubleshoot them. Failure at any stage can lead to bleeding complications or incomplete removal of stones.

## 1. Introduction

PCNL is a procedure of proficiency. For experienced urologists, it may be a day-in day-out routine procedure which is finished in an hour. But for a beginner, every step is of paramount importance. Negligence at any step can lead to catastrophic consequences causing severe bleeding, loss of tract, and failure of the procedure. PCNL has stood the test of time, and it has now evolved into a whole new era, where pyelolithotomy has become obsolete. Various modifications have come, but prone PCNL is the most common procedure performed for renal calculi today. However, there is paucity of articles dealing about the practical aspects of PCNL and troubleshooting during any difficulty. This article deals with the common challenges faced during PCNL.

## 2. Preoperative Planning

Analysing computed tomography (CT)/intravenous urography (IVU) imaging thoroughly is the most important first step in planning the procedure. Going through each and every film guides us for accurate puncture and approach to the calculi. Every case is different with reference to calyceal anatomy and the position of calculi. So, there are no rules for puncture. However, it is always better to enter via opposite calyx, if the stone is at poles. Pelvic stones are best managed by upper or middle calyx puncture. Lower pole puncture, though seems easy for newcomer, is the most unfriendly in PCNL usually owing to its anterior tilt.

## 3. Cystoscopic Placement of Ureteric Catheter

Usually 5 Fr or 6 Fr ureteric catheter is used. The catheter should be placed up to the upper calyx. This helps in keeping a limit to the medial extension of instruments during dilatation of tract under fluoroscopy. Mostly renal pelvic stones and even staghorn stones allow easy passage of ureteric catheter over guidewire. But, the impacted upper ureteric stones pose substantial difficulty in passage of guidewire. Getting caught in such a situation right in the beginning of the surgery is a very embarrassing situation, especially for a beginner.

Most of the times, simple manipulation of guidewire and ureteric catheter makes the passage into the renal pelvis. But, this should be done with extreme caution as there are high chances of perforating the wall of ureter and guidewire going out of the system. To prevent this untoward event, hydrophilic guidewire is always used for manipulation. Manipulation should be done in such a way that the guidewire passes first followed by ureteric catheter and never the opposite. Excessive force should be avoided. All these procedures should be done strictly under fluoroscopy.

Dislodging the stone using hydrostatic pressure through ureteric catheter with stone at the tip is another way that is helpful sometimes. Ureteroscope can be used to partially fragment and dislodge such stones into the renal pelvis. After difficult ureteric cannulation, the position of ureteric catheter position should be confirmed using contrast injection under fluoroscopy.

## 4. Initial Puncture/Access

**Figure 1:**
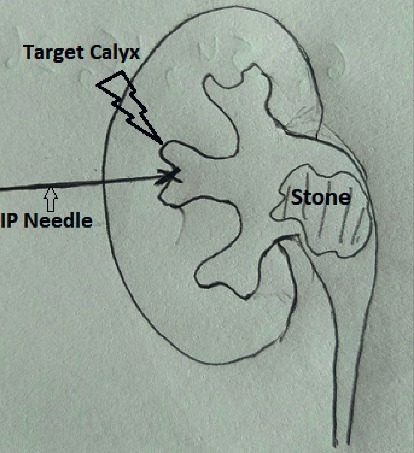
Identification of target calyx and the direction in horizontal axis in zero degree.

**Figure 2:**
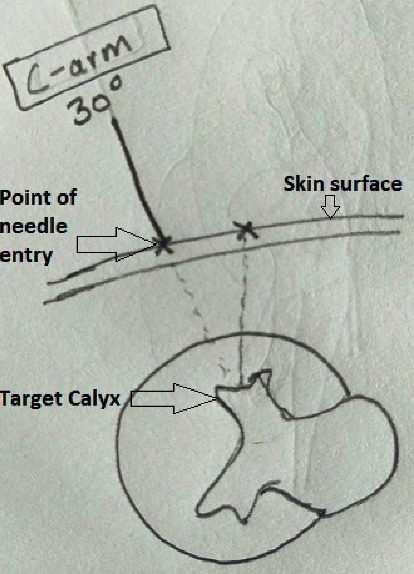
Site of entry and angle of entry to horizontal axis are decided in 30 degree.

**Figure 3:**
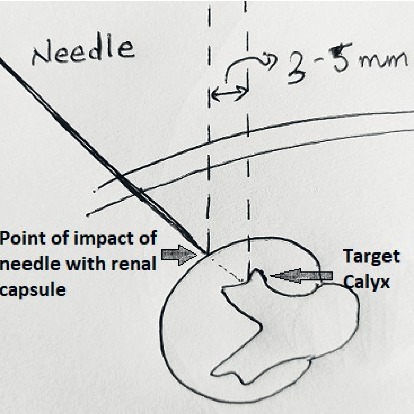
Desired point of entry (target calyx) is 3–5 mm medial to the needle tip at the point when kidney starts moving on impact of the needle.

After putting the patient in the prone position, the calyx for initial puncture is chosen. Contrast is injected through ureteric catheter to opacify the pelvicalyceal system. Contrast occupies the anterior calyx. However, the desired calyx is posterior one. Here comes the role of preoperative computed tomography film visualization, which helps in locating the relative position of posterior calyces in relation to the anterior calyces that are opacified. Upper and lower pole calyces are mostly compound calyces; hence, target calyx identification is relatively easy. The angle of calyx should preferably be in the direction of the calculi to prevent torque effect during fragmentation. Initial puncture is conventionally taught by Bull's eye technique or Triangulation technique. Combination of both techniques gives optimum results. The target calyx and the direction in horizontal axis are identified in zero degree (Figure 1). The site of entry and angle of entry are decided in 30 degree (Figure 2). The fluoroscopy is then placed in zero degree. The needle is advanced towards the kidney. The movement of kidney on fluoroscopy confirms the impact of needle to renal capsule. It is ensured that desired point of entry is 3–5 mm medial to the needle tip at this point (Figure 3). Further extension of the needle gives entry into the system. There is a feeling of give way on entry into the calyx if carefully appreciated. Many a times in staghorn calculus, the needle can be felt rubbing the stone directly.

**Figure 4:**
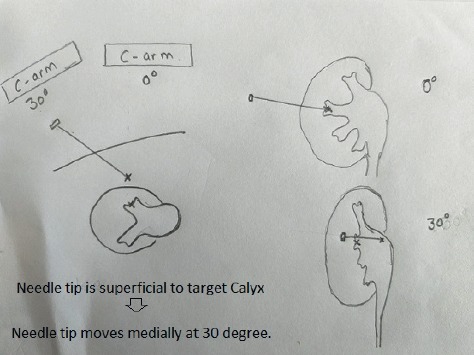
The needle tip moves medially, if superficial to the desired calyx when C-arm is moved to 30 degrees.

**Figure 5:**
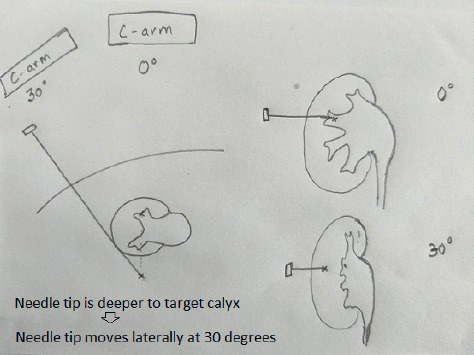
The needle tip moves laterally, if deeper to the desired calyx when C-arm is moved to 30 degrees.

If the puncture is difficult, 30 degree helps in depth assessment and evaluation of angle. When fluoroscopy is moved towards the urologist for 30 degrees, the needle tip moves medially, if superficial to the desired calyx (Figure 4), and it moves laterally if is deeper than the desired calyx (Figure 5). It is of utmost importance to enter via papilla of the calyx to prevent bleeding-related complications.

On withdrawal of stellate, free flow of urine can be seen. If urine is not coming freely, aspiration is done using 2 cc syringe and the needle is withdrawn slowly till urine starts coming. Sometimes, urine does not come despite good puncture, may be due to the nondilated system, or decompression of pressure through ureteric catheter or poorly functioning kidney. In such a situation, put the glidewire and confirm its position under fluoroscopy. If puncture is not done despite above efforts and there is contrast extravasation, aim for tip of ureteric catheter through upper calyx via zero and 30 degrees. Once position is confirmed by movement of tip of ureteric catheter by tip of needle, place the guidewire. Always remember “where there is a will, there is a way.”

There is one more situation when placement of ureteric catheter beyond the calculi is impossible. Here, first and foremost situation is anticipation. Study the CT and measure the distance of the most dilated part of pelvi-calyceal system from midline and the level of vertebra. Also measure the depth from the skin. Now first puncture into the system is done in direct vertical direction into the system. Urine flow on withdrawal of stellate confirms the position. Put contrast into the system and proceed in the way described above. If you have the facility of ultrasonography machine, then the job is relatively easier.

Recently, air is also used for opacification of the PCS. The identification of desired posterior calyx is easier in this method and hence puncture is accurate. The only practical problem is sometimes bowel gas hinders the visualization of PCS. Advising for proctocalyx enema one night prior and on the morning of surgery can reduce this problem. There is a small risk of air embolism reported in literature associated with it. [1].

## 5. Dilatation of Tract

It can be done by four methods: Alken metallic dilators, Amplatz fascial Teflon dilators, balloon dilators, and one shot dilatation. [2–5] Choice of dilators depends mainly on surgeon preference. Here, we are discussing mainly about metallic dilators. After placement of guidewire preferably in ureter or in the PC system, next crucial step is accurate placement of central rod. The incision is given along the guidewire up to the lumber fascia. Central rod is placed along the guidewire under Fluoroscopy guidance. Once entered into the system through calyx, direct the central rod towards the stone. This ensures further dilatation along the stone. Coaxial dilatation using metallic dilators is most effective. Metallic dilators are theoretically known to be traumatic, however being tougher it is more accurate for dilatation and it is better seen under Fluoroscopy. Sturdiness of metallic dilators should not be considered to be more traumatic to the adjacent structures. It is similar like a sharper and stronger knife is more accurate for surgery than a rough one. Teflon dilators are known to be safer, however amount of force required is more hence chances of over-shooting or under-dilatation are more. Balloon dilators are safest; however many a times it cannot dilate the tough tissues and it is non-reusable and hence expensive. It is advisable to never cross beyond the ureteric catheter medially during dilatation. At every dilatation good efflux of urine indicates proper dilatation. Next Amplatz sheath is placed over the dilator set used. Amplatz sheath is placed just beyond the dilators. Dilators are removed keeping the guidewire in place.

## 6. Fragmentation of Stones

Nephroscope is inserted into the sheath to visualize the system. Usually after dilatation there are some clots in the PCS which hinders the vision when Nephroscope is introduced. We can either push saline through ureteric catheter before introducing the Nephroscope that removes clot through the sheath. Or directly remove the clots under vision using forceps. Sometimes there is under-dilatation. In such a case, introduce the Nephroscope along the guidewire into the system and advance the Amplatz sheath gently over it. But this should be done with extreme caution. Otherwise, we have to dilate the system again up to the desired location using the method used earlier. In some cases there are few strands of tissue hindering the entry of Nephroscope, in such a case gentle tissue separation can be done using forceps.

Fragmentation of stones is done using pneumatic lithoclast or Lasers. Frankly speaking, in conventional PCNL, pneumatic lithoclast is more effective and faster in fragmenting the stones to the desired size and also there are less chances of injury to the wall of pelvis. Calculi seen are fragmented to the size preferably to the size bigger enough that can be grasped and removed using forceps. Dusting the stone cause their migration into other calyces. Smaller fragments are more cumbersome to hold as they fly away with irrigation and also it takes more time to remove them. To prevent migration of stones into other calyces, advance the Amplatz sheath gently up to the stone. This step should be done gently as it can tear the infundibulum. Any resistance felt should be taken with extreme caution.

After fragmentation inspect surrounding calyces for any residual fragments and also confirm the same under fluoroscopy. If stone is in inaccessible calyx, it can be brought under vision by jet of water through infant feeding tube inserted via Nephroscope. Otherwise, make direct puncture to the stone, and push it towards the pelvis using IP needle directly or injecting a jet of saline.

## 7. Placing a Double-J Stent

Place a glidewire by the side of ureteric catheter up to the urinary bladder and remove the ureteric catheter from below. DJ stent is then placed under vision and Fluoroscopy guidance. This avoids any contamination of urinary tract through the ureteric catheter part that is lying in unsterile area.

## 8. To Put or Not to Put a Nephrostomy

Next big question in every beginner's mind after finishing a PCNL is whether nephrostomy is needed? Bellman in 1997 first reported tubeless PCNL. [6, 7] Literature review has shown that tubeless PCNL is a safe and effective technique. Moreover, nephrostomy tube increases the postoperative pain, morbidity and duration of stay in comparison to tubeless PCNL. [8] But, still there are few indications which warrant a nephrostomy tube insertion after PCNL. Nephrostomy tube plays no role in preventing bleeding from the tracts. In today's world there are only two indications of nephrostomy: one if PCS has infection to stage the procedure, or second if the stone cannot be removed completely in single sitting and you want to stage the procedure. Simple compression of the loin after removal of Amplatz sheath for 3–5 minutes ensures adequate hemostasis. Rest can be done with a pressure bandage and injection of furosemide 20 mg. These maneuvers ensure clear urine after the procedure.

## 9. Conclusion

PCNL is considered as the “bread and butter” of urologists. But hands-on training for PCNL is still not adequate in most institutions during residency period. Hence, before starting independent practice, a urologist has to spend further 1-2 years in a high volume center to attain expertise in PCNL. Though PCNL is associated with little morbidity, its efficacy is unmatchable with other minimally invasive modalities. However, possible complications, such as bleeding, loss of tract can occur. Improved skills and modifications of the procedure may reduce the probability of adverse outcomes.

## Data Availability

No data is given in the article.
